# Sekundärmalignome nach Ganzkörperbestrahlung pädiatrischer Leukämiepatienten – eine kritische Re-Evaluation der multizentrischen ALL-SCT-BFM-2003-Studie

**DOI:** 10.1007/s00066-023-02070-0

**Published:** 2023-03-16

**Authors:** Christian Knaack, Michael Oertel, Hans Theodor Eich

**Affiliations:** Klinik für Strahlentherapie – Radioonkologie, Universitätsklinikum Münster, Münster, Deutschland

## Einleitung

Die Ganzkörperbestrahlung (TBI) ist eine wirksame Konditionierungsmodalität vor allogener Stammzelltransplantation (SCT) mit dem Ziel der Depletion des Knochenmarks und damit Eradikation der malignen Zellen [1]. Die multizentrische ALL-SCT-BFM-2003-Studie erzielte mit einer Kombination aus TBI und Etoposid ein gutes Langzeitüberleben für pädiatrische Patienten mit akuter lymphoblastischer Leukämie (ALL), unabhängig vom Donorstatus [2]. Die jetzt veröffentlichten Daten zu Sekundärmalignomen (SMN) erfordern eine kritische Re-Evaluation.

## Methodik

Es handelt sich um eine prospektive multizentrische Studie mit 705 Patienten. Die Daten wurden von September 2003 bis September 2011 sowie in der erweiterten Registrierung bis September 2013 erhoben. 558 Patienten erhielten hierbei eine Konditionierung mittels 12 Gy TBI als 2‑mal tägliche Radiatio von 2 Gy sowie Etoposid (60 mg/kg). Bei einem Kindesalter von bis zu 2 Jahren oder Kontraindikation gegen eine TBI (z. B. Vorbestrahlung des ZNS) erfolgte die Konditionierung mit Busulfan, Cyclophosphamid und Etoposid (110 Patienten). Die Nachbeobachtung fand über einen medianen Zeitraum von 5,3 Jahren statt (TBI: 5,8 Jahre vs. Non-TBI: 4,4 Jahre).

## Ergebnisse

Es wurden bei 33 Patienten insgesamt 39 SMN diagnostiziert; diese entfielen ausschließlich auf die Therapiegruppe mit TBI. Bezüglich der Entitäten wurden fünf Fälle eines myelodysplastischen Syndroms/einer akuten myeloischen Leukämie (13 %), 14 Fälle von Schilddrüsenkarzinomen (36 %), vier Glioblastome und Basalzellkarzinome (jeweils 10 %) sowie seltenere Erkrankungen berichtet. Bei sechs Patienten lagen mehrere SMN vor. Die 5‑ bzw. 10-Jahres-Inzidenzen von SMN betrugen 0,02 ± 0,01 und 0,13 ± 0,03. Nach einem medianen Follow-up von 5,8 Jahren nach Diagnose des SMN waren 64 % der Patienten noch am Leben, mit einer kumulativen Mortalitätsinzidenz infolge des SMN von 0,00 ± 0,00 nach 5 Jahren und 0,06 ± 0,02 nach 10 Jahren (bezogen auf die Gesamtkohorte). In der univariaten Analyse zeigte sich allein die TBI als signifikanter Risikofaktor für ein SMN (Testung von TBI, Alter bei SCT, Donortyp, Stammzellquelle, Remissionsstatus vor SCT, CMV-Konstellation, akute bzw. chronische „graft-versus-host disease“), während sich in der multivariaten Analyse kein Faktor als signifikant erwies. Eine vorherige ZNS-Bestrahlung war nicht mit dem Auftreten eines Glioblastoms assoziiert (*p* = 0,22).

## Schlussfolgerung der Autoren

Die Autoren kommen zu dem Schluss, dass der Goldstandard der TBI für pädiatrische ALL-Patienten weiterhin zu rechtfertigen ist, empfehlen aber prospektive, randomisierte Studien, um alternative Therapieschemata zu testen. Eine kritische Evaluation der TBI sollte insbesondere für Patienten mit Keimzellmutationen und Prädispositionssyndromen erfolgen.

## Kommentar

Die vorliegende Langzeitanalyse liefert wichtige Argumente zur Diskussion um den Stellenwert der TBI in der Behandlung pädiatrischer ALL-Patienten:

- Für Langzeitüberlebende der ALL nach TBI tragen SMN zur Mortalität bei und nehmen nach 5 Jahren kontinuierlich zu. Sie übersteigen jedoch nicht die rezidivbedingte Mortalität (auch nach 10 Jahren: ca. 2 × so hoch).
- Die Gesamtrate der SMN nach 5 Jahren liegt mit 2 % innerhalb des zu erwartenden Bereichs (5,3–5,8 %; Übersicht in [3]).
- Bei einem medianen Follow-up von 5,3 Jahren sollten die berichteten 10-Jahres-Raten der SMN vorsichtig interpretiert werden.
- Das Spektrum der SMN ist weit gefächert und meist adäquat behandelbar. Erkrankungen mit infauster Prognose wie das Glioblastom (4 Fälle) sind sehr selten.
- Die Kontrollgruppe ohne TBI ergab sich aus Patienten mit Kontraindikationen gegen die geplante TBI und Kindern bis 2 Jahren und ist daher kleiner (558 vs. 110) und weist signifikante Imbalancen auf (kürzeres Follow-up, Geschlecht, Alter, Donortyp, Stammzellquelle). Trotzdem überrascht die Rate von 0 % SMN im Chemotherapiearm, da therapiebedingte hämatologische SMN auch hier zu erwarten wären.
- Relevante Informationen wie die antileukämische Therapie vor Konditionierung sowie die Rate von Prädispositionssyndromen wurden nicht erhoben.
- Auch wurden zusätzliche chemotherapie- und bestrahlungsassoziierte Nebenwirkungen wie kardiopulmonale Toxizitäten nicht dezidiert thematisiert; diese sind jedoch relevant für Langzeitmorbidität und -mortalität [3, 4].
- In der FORUM-Studie konnte eine Konditionierung mit 12 Gy TBI und Etoposid bei ALL-Patienten gegenüber einer reinen Chemotherapiestrategie ein verbessertes Gesamtüberleben (91 % vs. 75 % nach 2 Jahren) bei gleichzeitig niedrigerer Rezidivrate und therapiebedingter Mortalität erzielen ([5]; Kommentar in [6]).
- Durch Weiterentwicklung der TBI hin zu modernen Bestrahlungsansätzen wie Total-marrow- bzw. Total-lymphoid-Bestrahlung, in denen anstelle des Ganzkörpers selektiv Knochenmark bzw. Lymphknoten adressiert werden, ist eine Reduktion von Toxizitäten zu erwarten [7]. Unklar ist hierbei, wie sich die Verwendung intensitätsmodulierter Bestrahlungstechniken mit entsprechendem Niedrigdosisbereich auf die Rate an SMN auswirkt.

### Schlussfolgerung

Eine Konditionierung mittels TBI ist einer reinen Chemotherapiekonditionierung bei pädiatrischen ALL-Patienten überlegen, jedoch mit einer (gering) erhöhten Rate von Sekundärmalignomen verbunden. Dies rechtfertigt keinen allgemeinen Verzicht auf die TBI, sondern sollte Ausgang für eine individualisierte Risikobewertung sein.

*Christian Knaack, Michael Oertel, Hans Theodor Eich, Münster*
